# Exploring Prostate Cancer Genome Reveals Simultaneous Losses of *PTEN*, *FAS* and *PAPSS2* in Patients with PSA Recurrence after Radical Prostatectomy

**DOI:** 10.3390/ijms16023856

**Published:** 2015-02-11

**Authors:** Chinyere Ibeawuchi, Hartmut Schmidt, Reinhard Voss, Ulf Titze, Mahmoud Abbas, Joerg Neumann, Elke Eltze, Agnes Marije Hoogland, Guido Jenster, Burkhard Brandt, Axel Semjonow

**Affiliations:** 1 Prostate Center, Department of Urology, University Hospital Muenster, Albert-Schweitzer-Campus 1, Gebaeude 1A, Muenster D-48149, Germany; E-Mail: prostata@uni-muenster.de; 2 Center for Laboratory Medicine, University Hospital Muenster, Albert-Schweitzer-Campus 1, Gebaeude 1A, Muenster D-48149, Germany; E-Mail: Hartmut.Schmidt-ZL@ukmuenster.de; 3 Interdisciplinary Center for Clinical Research, University of Muenster, Albert-Schweitzer-Campus 1, Gebaeude D3, Domagkstrasse 3, Muenster D-48149, Germany; E-Mail: voss@uni-muenster.de; 4 Pathology, Lippe Hospital Detmold, Röntgenstrasse 18, Detmold D-32756, Germany; E-Mail: u.titze@web.de; 5 Institute of Pathology, Mathias-Spital-Rheine, Frankenburg Street 31, Rheine D-48431, Germany; E-Mail: mahmoud.abbas@pathomail.de; 6 Institute of Pathology, Klinikum Osnabrueck, Am Finkenhuegel 1, Osnabrueck D-49076, Germany; E-Mail: joerg.neumann@pathomail.de; 7 Institute of Pathology, Saarbrücken-Rastpfuhl, Rheinstrasse 2, Saarbrücken D-66113, Germany; E-Mail: e.eltze@pathologie-saarbruecken.de; 8 Department of Pathology, Erasmus Medical Center, 's-Gravendijkwal 230, 3015-CE Rotterdam, The Netherlands; E-Mail: a.m.hoogland@erasmusmc.nl; 9 Department of Urology, Erasmus Medical Center, 's-Gravendijkwal 230, 3015-CE Rotterdam, The Netherlands; E-Mail: g.jenster@erasmusmc.nl; 10 Institute for Clinical Chemistry, University Clinic Schleswig-Holsteins, Arnold-Heller-Strasse 3, Haus 17, Kiel D-24105, Germany; E-Mail: Burkhard.Brandt@uksh.de

**Keywords:** prostate cancer, PTEN, PAPSS2, copy number variation, PSA recurrence, Affymetrix

## Abstract

The multifocal nature of prostate cancer (PCa) creates a challenge to patients’ outcome prediction and their clinical management. An approach that scrutinizes every cancer focus is needed in order to generate a comprehensive evaluation of the disease, and by correlating to patients’ clinico-pathological information, specific prognostic biomarker can be identified. Our study utilized the Affymetrix SNP 6.0 Genome-wide assay to investigate forty-three fresh frozen PCa tissue foci from twenty-three patients. With a long clinical follow-up period that ranged from 2.0–9.7 (mean 5.4) years, copy number variation (CNV) data was evaluated for association with patients’ PSA status during follow-up. From our results, the loss of unique genes on 10q23.31 and 10q23.2–10q23.31 were identified to be significantly associated to PSA recurrence (*p* < 0.05). The implication of PTEN and FAS loss (10q23.31) support previous reports due to their critical roles in prostate carcinogenesis. Furthermore, we hypothesize that the PAPSS2 gene (10q23.2–10q23.31) may be functionally relevant in post-operative PSA recurrence because of its reported role in androgen biosynthesis. It is suggestive that the loss of the susceptible region on chromosome 10q, which implicates PTEN, FAS and PAPSS2 may serve as genetic predictors of PSA recurrence after radical prostatectomy.

## 1. Introduction

Prostate cancer is the second most commonly diagnosed cancer in males worldwide [1]. It is a heterogeneous disease and its complexity presents a challenge that affects prediction of patients’ outcome and clinical management. A major feature of prostate cancer heterogeneity is the presence of multiple tumor foci within the prostate, which is observed in about 80% of radical prostatectomy specimens [2,3].

In recent years, there has been an upsurge of information that has expanded our knowledge of prostate cancer genomics. Genomic losses have been observed to occur more frequently when compared to genomic gains in prostate cancer [4,5]. From previous studies, androgens and the androgen signaling have been acknowledged to be crucial in the growth and progression of the prostate and prostate cancer [6,7]. Recognized chromosomal aberrations such as the TMPRSS2-ERG gene fusion, the gain of MYC, and the losses of TP53 and PTEN have been shown to be of prognostic value in prostate cancer [7,8,9,10,11]. The loss of PTEN is perceived to be a chromosomal event with translational impact, as it has been associated closely with recurrence of prostate specific antigen (PSA), prostate cancer progression and lethality [11,12]. Nevertheless a genomic prognostic marker is yet to be discovered and the major challenge in this quest is the observed heterogeneity of prostate cancer. In many cases, the tumors possess multiple cell populations with dissimilar genetic alteration profiles [2,13,14]. Presently, PSA recurrence following radical prostatectomy is used as a surrogate end-point for monitoring prostate cancer relapse [15].

In this study, 43 fresh frozen tumor foci and 10 non-tumor samples from 15 multifocal and 8 unifocal prostate cancers were investigated with the Affymetrix SNP 6.0 microarray tool (Affymetrix, Santa Clara, CA, USA). Copy number variation data was subsequently correlated to patients’ PSA recurrence status. Statistical significant associations are hoped to identify genes in altered chromosomal regions, whose biological relevance will reduce the unpredictability of prostate cancer’s progression.

## 2. Results and Discussion

With the Affymetrix Genotyping Console software (version 4.1.2, Affymetrix, Santa Clara, CA, USA), each chip experiment was quality controlled (QC) for contrast and intensity. All tumor foci (*n* = 43) show a QC range of 90.3%–98.9% with a mean QC value of 96.5%. For the non-tumor samples (nine blood samples and one benign prostate tissue (*n* = 10)), the QC range is between 96.8%–98.8%, with a mean QC value of 98.1%.

### 2.1. Chromosomal Copy Number Variation (CNV) Events

Genome-wide investigation of 43 prostate cancer specimens, nine blood samples and a single benign prostate tissue revealed copy number variation data in a total of 627 chromosomal regions in the tumor specimens and in 251 chromosomal regions in the benign tissue and blood samples. Comparison of CNV data from tumor and non-tumor samples enabled the identification and exclusion of matched CNVs, as they are recognized as potential germ-line CNV events. An observed frequency of distinct CNV events in 5 tumor foci was utilized as the minimum threshold frequency. The most frequent CNV gains were observed on chromosome regions: 22q11.1 (16/43; 37.2%), 16p12.1 (13/43; 30.2%), 15q22.31 (11/43; 25.6%), 9q21.11 (10/43; 23.3%), 8q21.11 (7/43; 16.3%) and 8q22.3 (7/43; 16.3%). The most frequent copy number losses were observed at 8p21.2 (21/43; 48.8%), 8p21.3 (21/43; 48.8%), 8p21.2–8p21.1 (19/43; 44.2%), 8p21.3–8p21.2 (18/43; 41.9%), 8p23.1–8p22 (15/43; 34.9%), 16q24.1 (14/43; 32.6%), 18q11.2 (14/43; 32.6%), 8p11.22–8p11.21–8p11.21 (13/43; 30.2%) and 10q23.31 (13/43; 30.2%). The genome-wide copy number variation results are summarized in Table 1. The most frequently observed chromosomal aberrations were copy number losses situated on the p arm of chromosome 8.

**Table 1:** Frequently observed copy number variation regions in investigated tumor foci. The gene *NKX3-1* is in bold because it is notable within the chromosomal region.

Copy Number Variation Regions	Number of Individual Tumor Foci (*n* = 43)	Genes Annotated to the Region
Gain		
22q11.1	16 (37.2%)	*OR11H1*, *POTEH*
16p12.1	13 (30.2%)	*GSG1L*
15q22.31	11 (25.6%)	*MEGF11*
9q21.11	10 (23.3%)	*ANKRD20A4*, *C9orf71*, *CBWD3*, *CBWD5*, *CBWD6*, *FAM122A*, *FOXD4L3*, *FOXD4L5*, *FOXD4L6*, *PGM5*
8q21.11	7 (16.3%)	*CRISPLD1*, *GDAP1*, *HNF4G*, *JPH1*, *LY96*, *MIR2052*, *PI15*, *RDH10*, *RPL7*, *STAU2*, *UBE2W*
8q22.3	7 (16.3%)	*ATP6V1C1*, *AZIN1*, *C8orf56*, *CTHRC1*, *DPYS*, *FZD6*, *GRHL2*, *KLF10*, *LRP12*, *MIR3151*, *NCALD*, *ODF1*, *SLC25A32*, *TM7SF4*
8q24.13	6 (14%)	*ANXA13*, *ATAD2*, *C8orf76*, *DERL1*, *FAM83A*, *FAM91A1*, *FBXO32*, *FER1L6*, *KIAA0196*, *KLHL38*, *MTSS1*, *NDUFB9*, *NSMCE2*, *RNF139*, *SQLE*, *TATDN1*, *TMEM65*, *TRIB1*, *TRMT12*, *WDR67*, *WDYHV1*, *ZHX1*, *ZHX1-C8ORF76*, *ZHX2*, *ZNF572*
Loss		
8p21.2	21 (48.8%)	*ADAM28*, *ADAMDEC1*, *BNIP3L*, *CDCA2*, *DPYSL2*, *KCTD9*, *NEFL*, *NEFM*, *NKX2-6*, ***NKX3-1***, *PNMA2*, *PPP2R2A*, *STC1*
8p21.3	21 (48.8%)	*ATP6V1B2*, *BIN3*, *BMP1*, *C8orf58*, *CHMP7*, *CSGALNACT1*, *DOK2*, *EGR3*, *EPB49*, *FAM160B2*, *FGF17*, *GFRA2*, *HR*, *INTS10*, *KIAA1967*, *LGI3*, *LPL*, *LZTS1*, *MIR320A*, *NPM2*, *NUDT18*, *PDLIM2*, *PEBP4*, *PHYHIP*, *PIWIL2*, *POLR3D*, *PPP3CC*, *R3HCC1*, *REEP4*, *RHOBTB2*, *SFTPC*, *SH2D4A*, *SLC18A1*, *SLC39A14*, *SORBS3*, *TNFRSF10A*, *TNFRSF10B*, *TNFRSF10C*, *TNFRSF10D*, *XPO7*
8p21.2–8p21.1	19 (44.2%)	*CHRNA2*, *EPHX2*, *PTK2B*, *STMN4*, *TRIM35*
8p21.3–8p21.2	18 (41.9%)	*ENTPD4*
8p23.1–8p22	15 (34.9%)	*C8orf48*, *CTSB*, *DEFB130*, *DEFB134*, *DEFB135*, *DEFB136*, *DLC1*, *FAM66A*, *FAM66D*, *FAM86B1*, *FAM86B2*, *FDFT1*, *KIAA1456*, *LONRF1*, *MIR3926-1*, *MIR3926-2*, *USP17L2*, *ZNF705D*
16q24.1	14 (32.6%)	*ATP2C2*, *C16orf74*, *COTL1*, *COX4I1*, *COX4NB*, *CRISPLD2*, *FAM92B*, *FOXC2*, *FOXF1*, *FOXL1*, *GINS2*, *IRF8*, *KCNG4*, *KIAA0182*, *KIAA0513*, *KIAA1609*, *MIR1910*, *MTHFSD*, *USP10*, *WFDC1*, *ZDHHC7*
18q11.2	14 (32.6%)	*ANKRD29*, *AQP4*, *C18orf45*, *C18orf8*, *CABYR*, *CHST9*, *HRH4*, *IMPACT*, *KCTD1*, *LAMA3*, *MIR320C2*, *NPC1*, *OSBPL1A*, *PSMA8*, *RIOK3*, *SS18*, *TAF4B*, *TTC39C*, *ZNF521*
8p11.22–8p11.21	13 (30.2%)	*ADAM2*, *ADAM18*
10q23.31	13 (30.2%)	*ACTA2*, *ANKRD22*, *ATAD1*, *CH25H*, *FAS*, *IFIT1*, *IFIT1B*, *IFIT2*, *IFIT3*, *IFIT5*, *KIF20B*, *KLLN*, *LIPA*, *LIPF*, *LIPJ*, *LIPK*, *LIPM*, *LIPN*, *MIR107*, *PANK1*, *PTEN*, *RNLS*, *SLC16A12*, *STAMBPL1*
10q23.2–10q23.31	10 (23.3%)	*PAPSS2*

### 2.2. Establishing Relationship between Copy Number Variation Regions and PSA Recurrence

**Figure 1:**
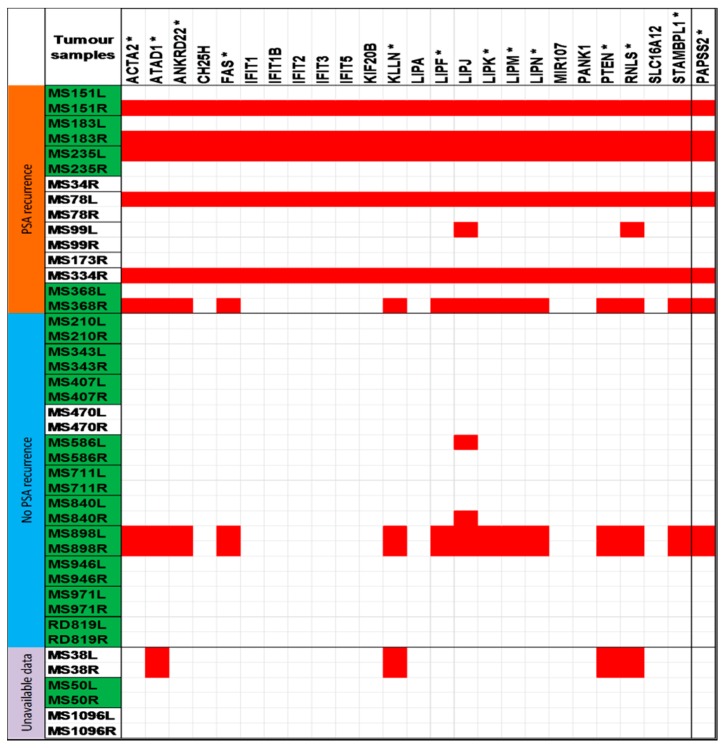
Summary illustration of genes with copy number losses located on chromosomes 10q23.31 and 10q23.2–0q23.31 (*PAPSS2*). Red shaded boxes indicate tumor foci with losses for specified genes. For tumor sample IDs, multifocal prostate cancers are highlighted in green and unifocal prostate cancers are not highlighted with color. In addition, the tumor foci were sub-categorized into groups based on the patients’ PSA recurrence status.

For 20 out 23 patients, PSA recurrence status was obtainable. Nine patients show PSA recurrence, while 11 patients show no PSA recurrence during 2.0–9.7 (mean 5.4) years of follow-up. Using the Fisher’s exact test to compare patients’ follow-up information and copy number variation data, specific locations on the chromosome 10q were identified as they showed correlation to PSA recurrence of prostate cancer. The copy number loss of 10q23.2–10q23.31 was identified as it correlated significantly to PSA recurrence (*p* < 0.05). Remarkably, the PAPSS2 gene was identified to be annotated to this region. The loss of the PAPSS2 gene was observed in 6 out of 15 tumor foci from patients with PSA recurrence and in one out of 22 tumor foci were obtained from patients without PSA recurrence (Table 2). However, adjacent to this interesting region is 10q23.31, which also exhibited a copy number loss. The loss of the 10q23.31 region did not correlate statistically with PSA recurrence (Table 2). Although on closer scrutiny, some distinct altered genes located on 10q23.31 were identified to correlate significantly with PSA recurrence (*p* < 0.05) (Supplementary Information Table S1). The genes identified on 10q23.31 were found to be variably altered in individual tumor foci and they include *ACTA2*, *ANKRD22*, *ATAD1*, *CH25H*, *FAS*, *IFIT1*, *IFIT1B*, *IFIT2*, *IFIT3*, *IFIT5*, *KIF20B*, *KLLN*, *LIPA*, *LIPF*, *LIPJ*, *LIPK*, *LIPM*, *LIPN*, *MIR107*, *PANK1*, *PTEN*, *RNLS*, *SLC16A12* and *STAMBPL1* (Table 1). As shown in Figure 1, Table 2 and Table S2, the altered genes: *ATAD1*, *KLLN*, *PTEN*, *RNLS*, *ANKRD22*, *LIPF*, *LIPK*, *LIPM*, *LIPN*, *ACTA2*, *FAS* and *STAMBPL1* were distinctly identified to be significantly associated to PSA recurrence (*p* < 0.05). They were noticeably altered in a significant number of tumor foci obtained from patients with PSA recurrence after surgical treatment. Of the genes residing in the 10q23.31 region, PTEN and FAS are considered to be the major players in prostate tumorigenesis. The PTEN and FAS genes were observed to be lost in 6 of 15 tumor foci from patients with PSA recurrence and in two out of 22 tumor foci from patients without PSA recurrence patients. In summary, the data obtained shows that the chromosomal regions 10q23.2–10q23.31 and 10q23.31 are frequently lost in prostate cancer and possibly more than one gene in the regions may be associated to PSA recurrence after radical prostatectomy.

**Table 2:** Summary of statistical correlation of copy number variation regions and patients follow-up data.

Regions with Copy Number Loss Genomic Position (Start–End) ^a^	Genes Implicated ^b^	PSA Recurrence	
		Yes (*n* = 9)	No (*n* = 11)
10q23.2–10q23.31 chr. 10: 89351602–89572982	*PAPSS2*	6/9 (66.7%) *	1/11 (9.1%)
10q23.31 chr. 10: 89572982–91817088	*ACTA2*, *ANKRD22*, *ATAD1*, *CH25H*, *FAS*, *IFIT1*, *IFIT1B*, *IFIT2*, *IFIT3*, *IFIT5*, *KIF20B*, *KLLN*, *LIPA*, *LIPF*, *LIPJ*, *LIPK*, *LIPM*, *LIPN*, *MIR107*, *PANK1*, *PTEN*, *RNLS*, *SLC16A12*, *STAMBPL1*	7/9 (77.8%)	3/11(27.3%)

^a^ Genomic position obtained from the Partek summary report with Refseq annotation; ^b^ Official gene symbols obtained from the Partek summary report with Refseq annotation; * Statistical significance using Fisher’s exact test (*p* < 0.05).

### 2.3. Discussion

The Affymetrix SNP 6.0 microarray tool kit was utilized to evaluate 15 patients with multifocal and 8 patients with unifocal prostate cancers. This investigation provided copy number variation data that exhibited the most frequent losses on 8p, 10q, 16q and gains on 8q. Chromosomal aberrations in these regions have been identified in previous studies and are compatible with our observations [16,17,18]. The copy number losses on chromosome 8p are the most recognized genetic variations in prostate cancer and this chromosomal feature is deemed the initiation site of prostate carcinogenesis [19]. The 8p region holds a large number of tumor suppressor genes; however it has been challenging to identify the specific “driver” of prostate tumorigenesis [20]. The most notable is the *NKX3-1* gene, which is located on 8p21.2 (Table 2). It is believed that NKX3-1 is crucial in the normal development of the prostate since it has been observed to be frequently lost in prostate cancer and PIN (prostatic intraepithelial neoplasia) lesions [21,22].

Due to the similarities of our genome-wide copy number variation data to earlier genomic study results, the goal of this study was to correlate copy number variation data to patients’ follow-up information (PSA recurrence status). In order to identify statistical correlations, all frequently altered chromosomal regions were utilized (Table 1 and Table S1). A statistical relationship was recognized between the loss of 10q23.2–10q23.31, the loss of unique genes residing on 10q23.31 and PSA recurrence (*p* < 0.05). Although the losses on chromosome 8p, 13q and 16q were observed so frequently (Table 1 and Table S1), they were not significantly linked to post-operative PSA recurrence. From the gene set (Table 2 and Figure 1), the following genes were identified distinctly to be associated with PSA recurrence: *PAPSS2*, *ATAD1*, *KLLN*, *PTEN*, *RNLS*, *ANKRD22*, *LIPF*, *LIPK*, *LIPM*, *LIPN*, *ACTA2*, *FAS* and *STAMBPL1*. The copy number losses of *PTEN*, *FAS* and *PAPSS2* were distinctly observed in the same unique tumor specimens (Figure 1). It is observed in prostate cancer that alterations such as deletions occur very often and span large genomic areas, thereby involving several genes. These genes, which are implicated in these susceptible areas may individually have prognostic implications. There is the need for these genes to be investigated further through functional analysis and comparative gene expression studies. However it is important that these genes are discussed and their biological functions briefly highlighted.

PTEN is the most recognized tumor suppressor gene in the region and is regarded to be a major player in prostate carcinogenesis [23]. The loss of PTEN has been strongly associated to PSA recurrence and progression of prostate cancer [12]. An astonishing observation was made by Haffner *et al.* [24], where they reported that PTEN is exclusively lost in distinct cancer lesions. They observed the loss of PTEN in distinct primary tumors and in seven evaluated metastatic sites. With this unique genetic profile, they suggested that the loss of PTEN indicated a monoclonal relationship between defined primary tumors and metastasis, and that the loss of PTEN may be a predictor of prostate cancer lethality. From these reports and in conjunction with our observation, PTEN is increasingly perceived to be a valuable predictive marker for prostate cancer. However, the use of PTEN as a prognostic indicator has been met with limitations due to its non-specificity to prostate cancer. It is well-known to be deleted in many human cancers [25,26].

Within the frequently deleted locus of 10q23.31 and its neighboring region 10q23.2–10q23.31, the genes FAS and PAPSS2 were identified to be lost. These genes are selected for discussions because of their biological functions in humans and their potential association with PSA recurrence. The aforementioned FAS gene belong to the tumor necrosis factor (TNF) family and encodes the FAS (Fatty acid synthase) receptor, which is an apoptosis initiator [27]. FAS triggers the apoptotic signaling by binding to the FAS ligand, since it is understood that FAS ensures that programmed cell death takes place in tissues. Therefore, the eventual loss of FAS, which is observed in several cancers is associated with reduced apoptosis, increased cellular proliferation and evasion of tumor cells [28,29]. PTEN and FAS have been reported to have an interactive relationship. It is described that PTEN regulates cellular survival and progression by inhibiting the phosphatidylinositol-3 kinase/Akt pathway, this it does by recruiting the apoptotic FADD (Fas-associated via death domain) mechanisms [30].

**Figure 2:**
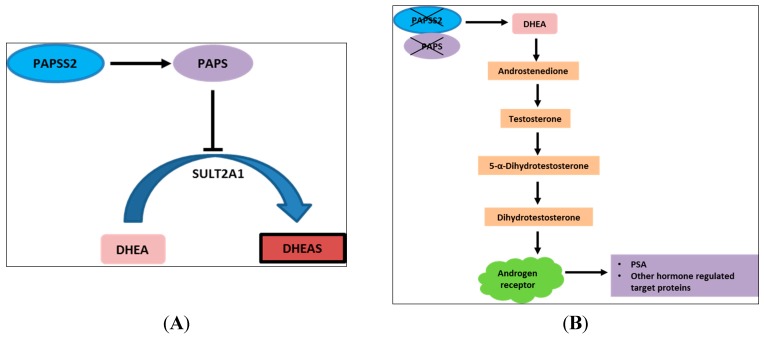
Hypothetical influence of PAPSS2 gene in PSA recurrence. The encoded PAPSS2 protein may have an effect on androgen biosynthesis and PSA recurrence. (**A**) PAPSS2 regulates the formation of PAPS, which subsequently regulates the conversion of excess DHEA to inactive DHEAS; (**B**) The absence of PAPSS2 and PAPS, results in the accumulation of DHEAS, formation of active androgens, activation of the androgen receptor. PAPSS2 = 3'-phosphoadenosine 5'-phosphosulfate synthase 2; PAPS = phosphoadenosine-phosphosulfate; DHEA = dehydroepiandrosterone; DHEAS = Dehydroepiandrosterone sulfate; SULT2A1 = DHEA sulfotransferase; PSA = prostate specific antigen. Adapted from [34,39].

From this study, the PAPSS2 (3'-phosphoadenosine 5'-phosphosulfate synthase 2) gene was solely annotated to the 10q23.2–10q23.31 region and also identified to correlate with PSA recurrence. Here, it is necessary to highlight the hypothetical effect of PAPSS2 loss on PSA recurrence. Its influence on PSA recurrence may be due to its role in the regulation of dehydroepiandrosterone (DHEA), which is a precursor for testosterone (Figure 2). PAPSS2 gene encodes the 3'-phosphoadenosine 5'-phosphosulfate synthase 2, which catalyzes the formation of PAPS (3'-phosphoadenosine-5'-phosphosulfate) via a 2-phase biosynthetic process. PAPS is a sulfate (SO_4_^2−^) donor that is required in many post-translational biological reactions [31] and actively involved in the sulfation conjugation of xenobiotic compounds [32]. The formation of PAPS from PAPSS2 utilizes inorganic sulfates and ATP (adenosine triphosphate) as substrates and it involves these phases: (1) reaction of inorganic sulfate and ATP to form APS (adenosine 5'-phosphosulfate) and (2) the subsequent reaction of APS with ATP to form PAPS [31,33,34]. Although the alteration of PAPSS2 gene has been clinically associated to Pakistani type of spondyloepimetaphyseal dysplasia (SEMD) [34] and colon cancer [35], it has not been shown to be strongly associated to prostate cancer. However, PAPSS2 has been reported to be poorly expressed in prostate cancer and may have a distinct role in prostate carcinogenesis [36]. Here we hypothesize that the copy number loss of PAPSS2 gene may lead to non-functional PAPSS2, which is unable to generate PAPS resulting in the accumulation of DHEA [34]. An increased level of DHEA is predicted to increase the rate of androgen biosynthesis and a potential increase of PSA levels. PSA is a target product of androgen receptor activity [37].

It was reported earlier that there may be two or more tumor suppressor genes in the 10q23–26 chromosomal region [38] due to the frequent loss of PTEN and its neighboring genes in prostate cancer [36]. With the implication of PTEN, FAS and PAPSS2 genes on 10q23.2–10q23.31 and 10q23.31, it is possible that the loss of these genes is critical in the pathogenesis of prostate cancer. PTEN has been studied frequently and is reported to possess important tumor suppressor roles, FAS is reported also to have pro-apoptotic functions and PAPSS2 is hypothetically a regulator of androgen biosynthesis. Their biological functions indicate that there may be a relationship between the genetic loss of these three genes PTEN, FAS, PAPSS2 and the PSA recurrence status of prostate cancer patients. The observed simultaneous loss of PTEN, FAS and PAPSS2 genes in prostatic tissues may have the potential of predicting PSA recurrence in patients after radical prostatectomy.

## 3. Experimental Section

### 3.1. Patients

Patients with multifocal and unifocal prostate cancer were treated with radical prostatectomy from 1998 to 2007 at the University Hospital Muenster, Germany and Erasmus Medical Center, Rotterdam, The Netherlands. Multifocal prostate cancer specimens were characterized with two or more tumor foci embedded in the prostate and the unifocal prostate cancer specimens were identified with a single tumor focus. The tumors were characterized by indicated clinical-pathological factors (Table 3). The age of the patients ranged from 50–71 years and the average patient age was 60 years at time of surgery. The pathological stage ranged from pT2 to pT4 and the Gleason score sum of individual tumor sections ranged from 6 to 9.

Following radical prostatectomy, PSA concentration in blood is expected to be below the lower limit of detection of the PSA assay. Since <0.1 ng/mL is the achievable lower limit of detection in modern PSA immuno-assays, all patients in this study were classified as having PSA recurrence if they had two consecutive PSA concentrations above 0.1 ng/mL, which progressed to higher PSA concentration during longer follow-up. Serum PSA measured before surgical treatment ranged from 3.68 to 45.2, with a mean of 12.5 ng/mL. Post-surgical serum PSA was measured twice yearly to evaluate PSA recurrence. Information on PSA recurrence was obtained from a follow-up period of 2.0–9.7 years, with a mean follow-up period of 5.4 years and a median follow-up period of 5.9 years. Nine patients had PSA recurrence during follow-up, 11 patients had no PSA recurrence and no PSA recurrence information could be obtained from three patients.

**Table 3:** Clinical-pathological parameters of prostate cancer patients.

Clinical Parameter	Status	*n* (%)
Age (years)	<60	10 (43.5%)
	>60	13 (56.5%)
Focality	Multifocal	15 (65%)
	Unifocal	8 (35%)
Pathological tumor stage (pT)	pT2	5 (21.7%)
	pT3	17 (73.9%)
	pT4	1 (4.35%)
Gleason score	≤7	22 (51.2%)
	>7	21 (48.8%)
PSA recurrence *	yes	9 (45%)
	no	11 (55%)

* Information on patients’ PSA recurrence status is available for only 20 out of 23 patients.

### 3.2. Clinical Specimens

Forty three fresh-frozen prostate cancer specimens and ten non-tumor samples (nine blood samples and one histological benign fresh-frozen prostate specimen) were obtained from 15 patients with multifocal prostate cancers and eight patients with unifocal prostate cancers. Two separate tumor foci were obtained from 15 multifocal prostate cancers (*n* = 30), while two tumor specimens were excised from different flanks of a large tumor from five unifocal prostate cancer cases (*n* = 10). In addition, single tumor foci were obtained from three unifocal prostate cancer cases (*n* = 3). The focality of the prostate cancer cases was determined from pathological reports, which contain topographical maps of locations of adenocarcinoma foci within the prostate gland [40,41]. The preparation and handling of tissue portions from the left and right side of the prostate have been previously described in Ibeawuchi *et al.* [13].

### 3.3. Quality Control and Data Analysis

Preparation of DNA and subsequent genome-wide analysis have been concisely described in Ibeawuchi *et al.* [13]. Each analyzed chip was quality controlled with the Affymetrix Genotyping Console software (version 4.1.2, Affymetrix, Santa Clara, CA, USA) [42]. Afterwards, intensity data as CEL files were exported for further analysis into the Partek^®^ Genomic Suite software (version 6.6) (Partek Incorporated, St. Louis, MO, USA) [43]. In the Partek Genomic Suite, all experimental CEL files were normalized against a Universal Reference (Hapmap 270 control samples). The Affymetrix 6.0 microarray chip contain more than 906,600 SNP (single nucleotide polymorphism) probes and these SNPs were exclusively interrogated for copy number variation (CNV) analysis by the genomic segmentation algorithm. To evaluate CNV, the genomic segmentation algorithm was utilized with the following parameters: minimum genomic markers/probes of 10, *p*-value of ≤0.001 and signal to noise ratio of 2 ± 0.3 (limits of detecting the normal range in a diploid region: 1.7 to 2.3). The Genomic segmentation algorithm [44] conducts its task of copy number analysis in two steps: Firstly, a breakpoint was established and secondly, the aberration status of that chromosomal region was ascertained. A breakpoint is recognized when a two-sided *t*-test statistically compares two neighboring regions/segments and there is a significant change in chromosomal abundance (*p* < 0.001). The aberration status of the region is thus established when a one-sided *t*-test was used to statistically compare the probe distributions mean of the chromosomal regions to the expected range of the normal (1.7 to 2.3). Annotations of significant regions were conducted with Refseq [45,46] and subsequent data handling was conducted with Microsoft^®^ Office Excel 2010 (Microsoft Corporation, Redmond, WA, USA).

### 3.4. Statistical Analysis

PSA recurrence status of patients was denoted with a “yes” or “no” based on serum PSA concentration levels during follow-up. Statistical associations of CNV altered regions and patients’ clinical-pathological data were evaluated by using Fisher exact test. *p*-values obtained in all tests were considered significant at *p* < 0.05. Statistical analysis was conducted using the Spotfire^©^ S+ 8.1 statistical software package (Tibco^©^ software Inc., Palo Alto, CA, USA).

## 4. Conclusions

We report that our copy number variation data reveals the loss of PTEN, FAS (10q23.31) and PAPSS2 (10q23.2–10q23.31) and these altered genes were found to be statistically associated to PSA recurrence. Although the exact influence of these genes on PSA recurrence is still unknown, they may be promising indicators to predict patients’ outcome. In addition, we draw attention to PAPSS2, which holds important biological functions that may increase our understanding of the biology of PSA recurrence and prostate cancer relapse. Based on the correlation between the copy number losses of PTEN, FAS, PAPSS2 and PSA recurrence and with the current quest for an improved biomarker, it is recommended that the molecular function and translational implication of PTEN, FAS, and PAPSS2 loss in prostate cancer are further investigated.

## Supplementary Materials

Supplementary materials can be found at http://www.mdpi.com/1422-0067/16/02/3856/s1.
